# Decoding *Collimonas pratensis* PMB3(1) responses during biotite interaction and dissolution: a multi-omics and geochemical perspective

**DOI:** 10.1128/aem.00704-25

**Published:** 2025-09-19

**Authors:** Laura Picard, Marie-Pierre Turpault, Jean Armengaud, Stéphane Uroz

**Affiliations:** 1 INRAE, IAM, Université de Lorraine 137665, Nancy, France; 2 BEF, INRAE 27057, Nancy, France; 3 CEA, Département Médicaments et Technologies pour la Santé (DMTS), Université Paris Saclay, INRAE 27057, Bagnols-sur-Cèze, France; Washington University in St. Louis, St. Louis, Missouri, USA

**Keywords:** mineral weathering, nutrient-poor soils, *Collimonas*, forest, transcriptomics, geochemistry and mineralogy

## Abstract

Mineral weathering bacteria are known to mobilize the nutrients entrapped in minerals using acidification- and chelation-driven mineral weathering (MWe) mechanisms. Through these mechanisms, bacteria are expected to play important roles in soil nutrient cycling and tree nutrition. Among the most effective MWe bacteria identified, *Collimonas* are particularly interesting due to their occurrence in the rhizosphere and their ability to acidify their environment using a glucose methanol choline oxidoreductase and to mobilize iron using malleobactin. Until now, the regulations of these functions according to nutrient availability and presence/absence of minerals remain uncharacterized, as does the potential involvement of other direct and indirect MWe mechanisms. In this context, we investigated how the solution chemistry and the functions expressed by strain PMB3(1) were regulated according to the concentrations of iron and magnesium and the presence/absence of biotite. Transcriptomics and proteomics dual approach highlighted that under nutrient-depleted conditions and in the absence of biotite, strain PMB3(1) increased the expression of genes and abundance of proteins related to osmoprotection, stress, and iron mobilization. On the contrary, in the presence of biotite, an upregulation of genes and proteins associated with surface sensing, motility, chemotaxis, transport, acidification, and iron storage was observed. These new findings represent a crucial first step in understanding the regulatory processes and mechanisms employed by *Collimonas* during its interaction with and weathering of biotite. They show that bacteria respond to nearby minerals in more complex ways than simply reacting to a lack of nutrients.

**IMPORTANCE:**

Minerals and rocks represent reactive interfaces at the geochemical level and a particular habitat at the microbial level. They are, however, usually considered inert substrata, although they represent 80% of the soil composition. The works performed on interactions between minerals and bacteria have mainly considered anoxic processes and microorganisms and poorly soil heterotrophs. In this context, our understanding of the role of soil minerals and rocks in soil fertility and the relative contribution and the molecular mechanisms employed by effective mineral weathering bacterial communities remain poorly documented. The combined use in our study of transcriptomics, proteomics, and geochemical analyses permitted filling this gap. The new findings obtained here suggest that minerals impact the global metabolism and the effectiveness at weathering of the strain *Collimonas pratensis* PMB3(1). They also reveal that the behavior exhibited by this bacterial strain extends beyond a mere reaction to the lack of nutrients. The complex interactions occurring between the physicochemical properties of these minerals and the activities of the MWe bacteria we observed *in vitro* offer a new view of the relative importance of minerals and rocks in *in situ* processes (e.g., nutrient cycling, soil fertility, and tree nutrition) at both geochemical and biological levels.

## INTRODUCTION

In temperate regions, forest ecosystems mainly grow on soils with low nutrient availability, often very acidic or basic, and rocky, the fertile soils being dedicated to agriculture. Soils of forest ecosystems are in addition characterized by an absence of fertilization (nitrogen, phosphorus, and potassium [NPK]), a very rare practice for forest ecosystems compared to agricultural systems. In this context, the nutritive elements required for the nutrition, the growth of the trees and their microbial associates as well as for the long-term functioning of forest ecosystems come from atmospheric deposits, the recycling of the nutrients coming from organic matter (OM) decomposition or from dissolution of the minerals and rocks (i.e., mineral weathering [MWe]) (1–4). Rocks and minerals are consequently important contributors to soil fertility, but in acidic conditions, the remaining minerals are poorly weatherable, as is the case with phyllosilicates (e.g., biotite). Forests must therefore rely on their own resources, and on the long term, the decline in soil fertility caused by soil aging (i.e., physical, chemical, and biological modification of soil along time) can lead to environmental regression, marked by smaller trees with slower growth and important modification of the soil microbiota (5, 6). This phenomenon is exacerbated in managed forests, where wood biomass export strongly contributes to the impoverishment of the soil (7–9). Understanding how trees maintain their growth in such low fertility conditions and what the mechanisms are, and the contribution of soil microorganisms to nutrient mobilization and tree nutrition are key questions.

While plants strongly condition the soil microbiota occurring outside and inside their root system, soil conditions and especially nutrient availability are also recognized key factors (9–11). Indeed, whatever the soil compartments considered (i.e., organic matter, minerals/rocks, aggregates, rhizosphere), microorganisms have to cope with variable physicochemical conditions. Large-scale studies have clearly evidenced how the structure and taxonomic composition of soil microbial communities depend on pH, C and N content, concentrations of phosphorus and base cations (12–17). However, few studies have investigated both the effect of plant and soil conditions, as it requires considering the same plant growing in soils with similar pedoclimatic conditions (e.g.*,* chronosequence).

Members of the rhizosphere soil microbiota (i.e., symbiotic fungi, bacteria) typically present particular functional traits poorly represented in the surrounding bulk soil, such as the ability to mobilize nutrients (e.g., P, Fe, K, Mg), to access water, and to promote plant growth (18–22). Noticeably, this functional enrichment has been shown to vary according to the plant species, the soil conditions, but also according to the function considered (23–25). Among these functional communities, effective MWe bacteria are more abundant in the soil horizons and soil types characterized by lower nutrient availability (i.e*.*, lower base cation concentrations) as compared to conditions with higher nutrient availability, suggesting that nutrient availability is an important driving factor (13, 26, 27). The manipulation of the concentration of available K or Mg in a soil naturally poor in these elements supported this hypothesis, revealing that a low input of these elements induced a rapid decrease in the frequency of effective MWe bacteria and particularly of the collimonads (28). While the functional roles of the rhizosphere microbiota have been demonstrated, our understanding of the mechanisms employed, their conservation among bacteria, and the potential regulatory mechanisms remains under-investigated.

The ability to weather mineral is broadly distributed among different taxa such as *Bacillus*, *Burkholderia*, *Caballeronia, Pantoea,* or *Pseudomonas*, showing that this function is not a specific trait associated with a single taxonomic group. While a high variability has been shown for different genera collected in forest soils, a good conservation of the MWe function was observed for bacteria belonging to *Collimonas,* making representatives of this taxon interesting models (29, 30). Considered as members of the rare biosphere based on soil metagenomic analyses, collimonads can be found paradoxically dominant in particular habitats, such as in the mycorrhizosphere and the mineralosphere (31, 32). Their presence in nutrient-poor and acidic soils with limited human disturbance associated with the presence of fungi suggests a particular ecology and an adaptation to oligotrophic conditions (29, 33–35). The combination of mutagenesis and (geo)chemical analyses has permitted better understanding of the molecular mechanisms used and to identify the corresponding genes used by strain *Collimonas pratensis* PMB3(1) to effectively weather minerals. This strain is capable of weathering minerals through (i) an acidification-driven mechanism, using a glucose/methanol/choline (GMC) oxidoreductase that converts glucose into protons and gluconic acid (36) and (ii) a chelation-driven mechanism, based on the production of a siderophore (i.e., malleobactin) (37). While the mechanisms and genes seem conserved among collimonads (36, 37), the regulatory mechanisms used in the presence of complex minerals remain unknown, as the potential implication of other mechanisms related to mineral interaction, sensing, colonization, weathering, or related to physiological adjustments.

In this context, this study aimed to determine which molecular mechanisms and regulatory mechanisms are employed by strain PMB3(1) of *Collimonas pratensis* when incubated in nutrient-depleted conditions with and without a mineral frequently found in acidic forest soil (i.e*.*, biotite). This bacterial strain was considered a model organism due to its ecological origin (38), its high effectiveness at weathering (35), at promoting plant growth (39), and because some of the molecular mechanisms conferring the MWe ability to this strain have already been identified (36, 37), but not their regulation. All these features suggest that the strain PMB3(1) of *Collimonas pratensis* is particularly adapted to a nutrient-poor environment and interaction with minerals. In this study, we compared different treatments to investigate the response in terms of upregulated genes and more abundant proteins of this strain to (i) conditions depleted or not depleted in Mg and Fe (two elements contained into rocks and minerals) and (ii) the presence/absence of a mineral (i.e., biotite) carrying these nutrients incubated in the depleted or nondepleted conditions. To answer these questions, we combined (geo)chemical, transcriptomics, and proteomics analyses to characterize the activated or repressed processes.

## RESULTS

### Chemical and biological analyses of the solution

The chemical analyses evidenced how the solution chemistry was impacted by our amendment in Fe and Mg, the introduction of biotite, and the activity of strain PMB3(1).

First, these analyses confirmed the deficiency of Mg and Fe in the nutrient-depleted treatments (i.e., B, **B**Hm medium devoid of Fe and Mg; BP, **B**Hm medium devoid of Fe and Mg inoculated with the strain **P**MB3(1); and BBP, **B**Hm medium devoid of Fe and Mg with **B**iotite and inoculated with the strain **P**MB3(1)), and on the contrary, their presence in the complete treatments (i.e., BC, **B**Hm medium **C**omplete (containing Fe and Mg) and BCP, **B**Hm medium **C**omplete (containing Fe and Mg) with the strain **P**MB3(1)). In the abiotic treatments, the introduction of biotite allowed for a low, but significant increase in Fe (from 0.06 to 0.19 mg.L^−1^), Mg (from 0.03 to 0.22 mg.L^−1^), and Al (from 0.01 to 0.05 mg.L^−1^) in the **B**Hm medium devoid of Fe and Mg with **B**iotite (BB) compared to the culture medium alone (B) (*P* < 0.05) (Table 1). This increase was explained by the passive dissolution of biotite, a phenomenon known to occur when freshly prepared mineral surfaces are introduced in an aqueous solution.

**TABLE 1:** Solution analyses^*a*^

Conditions				Chemical analyses					
Treatment	Medium	Biotite	Bacteria	pH	Gluconate	Ca	Mg	Al	Fe
B	−Fe, −Mg	−	−	6.32 ± 0.00^a^	0	3.08 ± 0.19^a^	0.03 ± 0.00^a^	0.01 ± 0.02^a^	0.06 ± 0.00^a^
BB	−Fe, −Mg	+	−	6.33 ± 0.01^a^	0	4.88 ± 0.11^b^	0.22 ± 0.00^b^	0.05 ± 0.03^a^	0.19 ± 0.05^b^
BC	+Fe, +Mg	−	−	6.32 ± 0.00^a^	0	3.48 ± 0.22^a^	14.20 ± 0.1^c^	0.01 ± 0.00^a^	0.88 ± 0.05^c^
BP	−Fe, −Mg	−	+	3.01 ± 0.01^b^	1122 ± 121^a^	3.50 ± 0.02^a^	0.04 ± 0.00^a^	0.01 ± 0.00^a^	0.13 ± 0.01^b^
BBP	−Fe, −Mg	+	+	3.26 ± 0.01^c^	711 ± 73^b^	6.65 ± 0.57^c^	0.37 ± 0.01^d^	0.18 ± 0.00^b^	0.49 ± 0.00^d^
BCP	+Fe, +Mg	−	+	3.07 ± 0.01^b^	1220 ± 35^a^	3.52 ± 0.01^a^	14.41 ± 0.0^c^	0.06 ± 0.02^a^	0.93 ± 0.02^c^

^*a*^ For each element, the values with different superscript letters (a, b, c, and d) are significantly different according to one-factor (treatment) ANOVA and a Bonferroni-Dunn test (*P* < 0.05). B, control medium deprived in Mg and Fe without bacteria; BB control medium deprived in Mg and Fe with biotite and without bacteria; BC, complete medium containing Fe and Mg without bacteria; BP, medium deprived in Mg and Fe with bacteria; BBP medium deprived in Mg and Fe with biotite and with bacteria; BCP, complete medium containing Fe and Mg with bacteria. In the Biotite or Bacteria column, + and − indicate presence and absence, respectively.

The inoculation of strain PMB3(1) significantly increased the dissolution level of biotite compared to the abiotic treatments (BBP >BB), as stated by the concentrations measured in the solution for Fe (0.49 mg.L^−1^ with PMB3(1) vs 0.19 mg.L^−1^ in absence), Al (0.18 mg.L^−1^ with PMB3(1) vs 0.05 mg.L^−1^ in absence), and Mg (0.37 mg.L^−1^ with PMB3(1) vs 0.22 mg.L^−1^ in absence), all nutrients absent from the culture medium and only present in biotite (Table 1). The activity of strain PMB3(1) was also visible due to pH changes, with BP (medium devoid of Fe and Mg) and BCP (complete medium containing Fe and Mg) treatments generating the most acidic conditions (pH = 3) compared to the BBP treatment (medium devoid of Fe and Mg with biotite; pH = 3.26) and the non-inoculated treatments (B, BB, and BC; pH = 6.32) (Table 1). Noticeably, these variations of pH were associated with a significant variation in the concentration of gluconic acid. Gluconic acid was the only organic acid detected, and its concentration varied in the solution from 711 mg.L^−1^ in BBP treatment to 1,200 mg.L^−1^ in the BP and BCP treatments (Table 1). The dilution/plating procedure revealed that the density of bacteria present in the solution was similar between the treatments, with a minimum of 1.5 × 10^7^ cell.mL^−1^ in the BCP treatment, 3 × 10^7^ cell.mL^−1^ in the presence of biotite (BBP), and a maximum of 9.3 × 10^7^ cell.mL^−1^ in the absence of biotite (BP). Inspection of the biotite particle surfaces after removal of the liquid phase in the BBP treatment with a microscope did not allow visualization of any biofilm; only some isolated cells were visible on biotite particles. A simple addition of sterile water removed these cells, evidencing that there was no development of a strong interaction between mineral particles and bacterial cells (i.e., biofilm) in our experimental conditions.

### Transcriptome and proteome statistics

Incubation was stopped after 20 h for five reasons: (i) to be in conditions where mineral weathering is quantifiable and greater than passive dissolution, (ii) to have still glucose to metabolize for bacteria (to maintain active the acidification-driven weathering mechanism), (iii) to let the bacteria in nutrient-depleted conditions (i.e., absence or low availability of Fe and Mg), (iv) to limit precipitation events, and (v) to have enough cell biomass for RNA and protein analyses. Preliminary experiments highlighted that a 20 h incubation time was adapted to fit with these requirements. This time point has been considered as an early time in the interaction between mineral and strain PMB3(1).

For the RNA-based analyses, the number of reads retained after barcode removal and quality control varied from 16 to 23 million (235 million in total) per sample, giving a total of 70 Gb of 150 bp paired-end sequence data. After removal of unmapped sequences and remaining rRNA sequences, 78% to 95% of the total number of reads were retained for further analyses. Considering the differentially expressed genes (DEGs) with a significant adjusted *P* value and a minimum of 50 reads in one of the treatments, our analyses revealed: (i) 196 DEGs for BP vs BCP (depleted vs rich media, without biotite), (ii) 3,287 for BP vs BBP (medium devoid in Fe and Mg without or with biotite), and (iii) 3,097 for BBP vs BCP (medium devoid of Fe and Mg with biotite vs complete medium with biotite). When a threshold of +0.9/–0.9 log2FC was applied, DEG number reached 116 (BP vs. BCP, corresponding to 2.1% of the genes of strain PMB3(1)), 2,512 (BP vs. BBP, corresponding to 39.2% of the genes of strain PMB3(1)), and 2,400 (BBP vs. BCP, corresponding to 37.6% of the genes of strain PMB3(1)). For the protein-based analyses, a total of 2,275,827 MS/MS spectra that were recorded at high resolution, among which 1,316,391 (57.8%) were assigned to peptide sequences attributed to strain PMB3(1) proteins. On average, 36,661 peptide sequences were identified per sample. Based on stringent parameters (at least two peptides per protein and FDR below 1%), a total of 2,447 (45% of the theoretical proteome of strain PMB3(1)) proteins have been identified. Considering the differentially abundant proteins (DAPs) with a minimum of 30 reads in one of the treatments, a significant adjusted *P* value and a threshold of +0.5/−0.5 log2FC, our analyses revealed: (i) 29 DAPs for BP vs. BCP, (ii) 56 for BP vs. BBP, and (iii) 52 for BBP vs. BCP.

**Fig 1:**
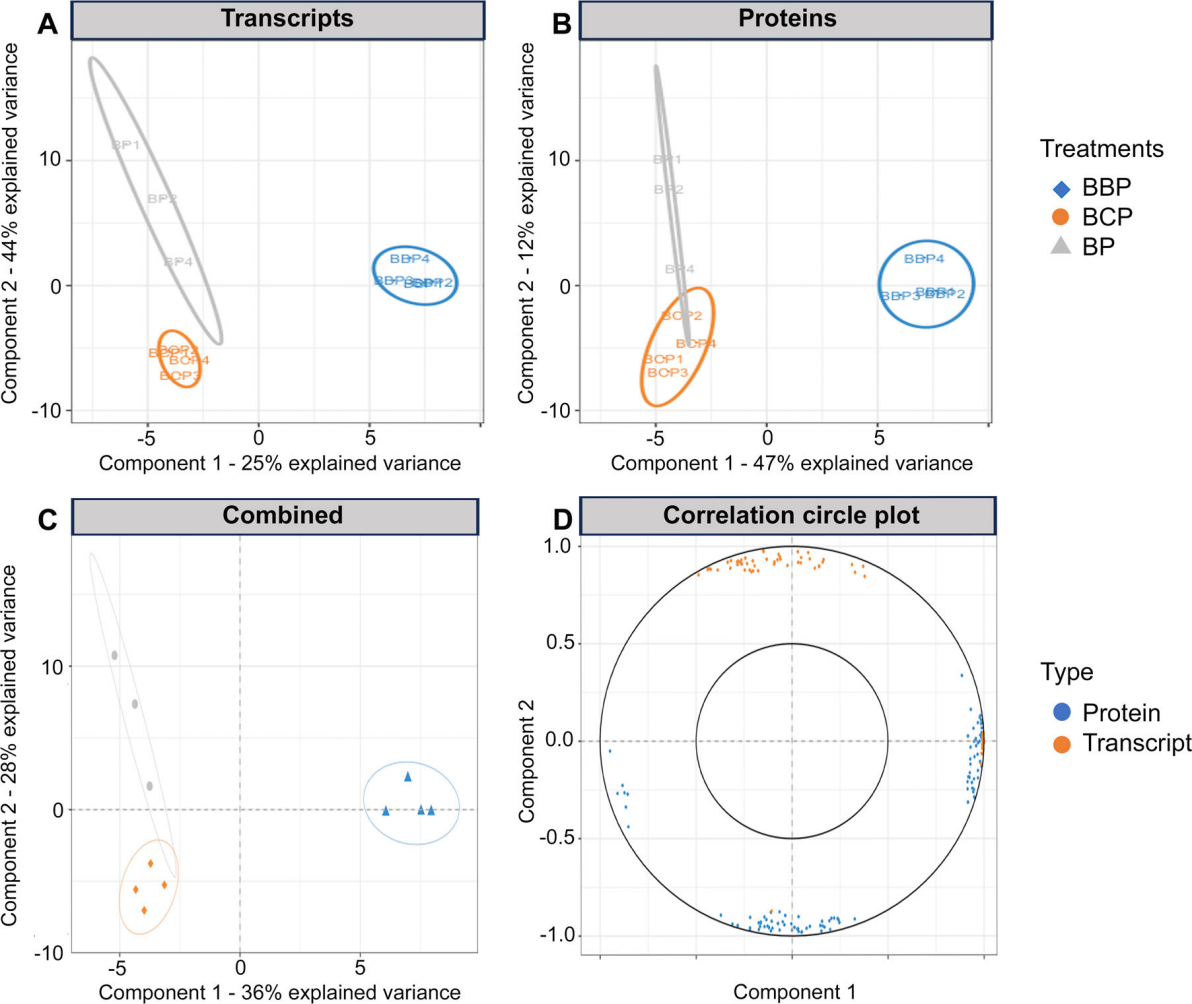
Treatment (BP vs. BBP vs. BCP) comparison based on multivariate analyses of the transcriptome and proteome data. Visualization of sPLS sample projection plots according to (A) RNAseq data, (**B**) proteomics data, and combined (RNAseq +Proteomics) data (**C**). In each panel (**A–C**), the color code is as follows: BP, gray triangles; BCP, orange circles; BBP, blue diamonds. Panel **D** presents a correlation circle plot highlighting the 100 genes the most associated with the treatments considered, that is, BP (nutrient-poor condition without biotite), BCP (nutrient-rich condition without biotite), and BBP (nutrient-poor condition with biotite). The color code is as follows: protein (blue) and transcript (orange). Confidence ellipses for each treatment are plotted to highlight the strength of the discrimination (confidence level set to 95%).

The global analysis done using MixOMICs (through a sparse partial least squares approach; sPLS) allowed the visualization of the separation of the BBP treatment (where biotite was introduced) from the other treatments (i.e., BP and BCP) and thus, whatever the method used (RNA or protein based), but also when RNA- and protein-based analyses were combined (Fig. 1). This first analysis was completed by a DESeq2 analysis applied on the different treatment comparisons. The trends observed appeared similar, although the RNA-based approach provided many more entities whose expression is regulated than the protein-based approach.

**Fig 2:**
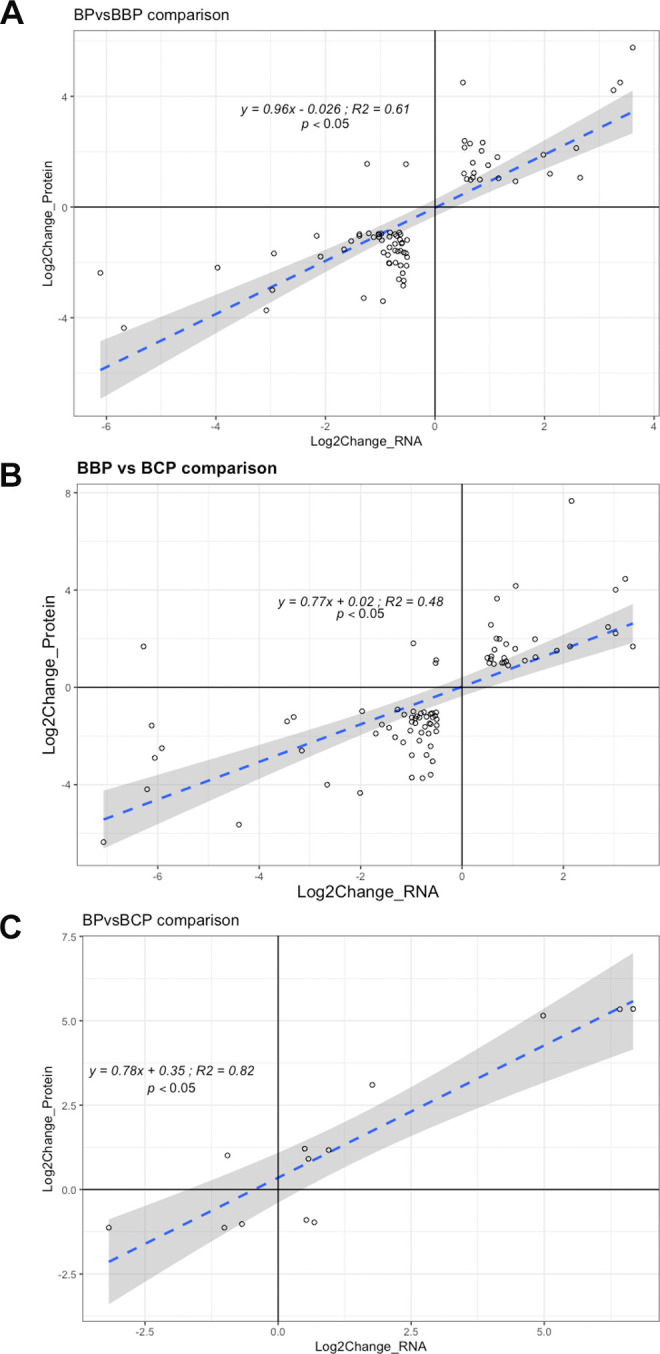
Relationship between the transcriptomic and proteomic data for the different treatment comparisons. (**A**) Relationship between DEG and DAP in the treatments BP vs. BBP. (**B**) Relationship between DEG and DAP in the treatments BBP vs. BCP. (**C**) Relationship between DEG and DAP in the treatments BP vs. BCP. Linear regression analyses have been done for each comparison, and the R^2^ and *P* values are presented.

When comparing the DEGs and DAPs, our analyses revealed similar patterns of regulation for the genes in common for the same treatment comparison ((i) 42% for the comparison BBP vs BCP; 45% for the comparison BP vs. BBP), supported by significant correlations (*P* < 0.0001; R^2^: 0.48 for BBP vs. BCP; R^2^: 0.61 for BP vs. BBP). All the genes in common were recovered for the comparison BP vs BCP, but corresponded to only 12 genes (R^2^: 0.82 for BP vs BCP) (Fig. 2). Genes that were differentially regulated (according to the log2FC threshold used for each method) and common to the RNAseq and proteomics data set are presented in Table S1.

### Clusters of orthologous groups (COG) category variations according to the treatments

The analysis of the DEG/ DAP of strain PMB3(1) in terms of clusters of orthologous groups revealed differences that can be attributed to the analytical methods (transcriptomics vs proteomics) and others to the treatments (BP vs BCP vs BBP) (Fig. S2). Noticeably, the patterns observed in proteomics differed from the transcriptomics ones, a difference explained by the different sequencing depth of the two methods, the low number of DAPs compared to DEGs (e.g., for the BP vs. BBP comparison, 2,192 DEGs vs. 112 DAPs), and the assignation of the related genes in uncharacterized COG groups. When considering the effect of depletion in Mg and Fe (BP vs. BCP), the assignment of DEG/ DAP of strain PMB3(1) into the clusters of orthologous groups categories, the main differences at transcriptomic level corresponded to poorly characterized groups (S, R) and to the D group (cell cycle control and division) both with a higher relative abundance in the BP treatment (BP >BCP), while the T group (signal transduction mechanisms) was higher in BCP. At the protein level, the M (cell membrane biogenesis), P (inorganic ion transport and metabolism), and C (energy production) groups presented a higher relative abundance in BP (BP >BCP) and, on the contrary, the group E (amino acid transport and metabolism) was higher in BCP. When considering the effect of nutrient availability (BCP vs. BBP), the main differences at the transcriptomic level corresponded also poorly characterized groups (S, R) and to signal transduction (T) and transcription (K) both with a higher relative abundance in the BCP treatment, while the J group (translation ribosomal structure and biogenesis) was higher in the BBP treatment. No difference was observed at the protein level for the main COG categories detected. When considering the effect of the presence/absence of biotite in the culture medium (BP vs BBP), the assignment of DEG/ DAP of strain PMB3(1) into the clusters of orthologous groups categories revealed an increase of the R (poorly characterized), K (transcription), O (post-translational modification, protein turnover, chaperones), and T (signal transduction) groups in the BP treatments and, on the contrary, an increase of the J (translation ribosomal structure and biogenesis) group in the BBP treatment. At the protein level, most of the COG categories presented a high relative abundance in the BBP treatment.

### A limited effect of the depletion of Fe and Mg in the culture medium

The BHm medium was used in our study as a minimal culture medium for two reasons: (i) its chemical composition fits relatively well with the chemistry of the soil (as previously determined (40) from which strain PMB3(1) was isolated, and (ii) it presents a low buffer capacity adapted to investigate the acidification-driven mineral weathering mechanisms employed by strain PMB3(1). A modified version of this medium devoid of iron (Fe) and magnesium (Mg) was used for treatments B, BP, or BBP. This choice was based on the hypothesis that limiting concentrations of Fe and Mg may induce a response of the effective MWe strain PMB3(1) considered. The comparison of the BP (i.e., medium devoid of Fe and Mg) and BCP (i.e., medium with no limitation) treatments permitted testing this hypothesis. Both RNA- and protein-based analyses revealed a very small number of genes differentially regulated between the BP and BCP treatments, with only 116 DEGs and 29 DAPs identified in the transcriptome and proteome analyses, respectively. Among them, 89 DEGs and 18 DAPs were upregulated in the treatment deprived of Mg and Fe (BP) (Table 2). In the BP treatment, the main genes upregulated corresponded to cations, heavy metals, and magnesium transport systems (i.e., NKI69999.1 to NKI70001.1, and NKI68856.1) as well as to iron homeostasis (i.e., siderophore transport (NKI72010.1), mobilization of iron from bacterioferritin (NKI72009.1)). These analyses also revealed upregulation in the BP treatment (BP >BCP) of three proteins with a TaT signal: (i) a formate dehydrogenase-O (NKI67910.1; log2FC = −0.97), (ii) a Ser/Thr protein phosphatase (NKI69616.1; log2FC = −0.86), and (iii) the small subunit of the GMC oxidoreductase (NKI70794.1; log2FC = −1.07). Noticeably, this GMC oxidoreductase is the main enzyme employed by *Collimonas* during the acidification-driven mineral weathering mechanism (36).

**TABLE 2:** List of the DEGs between the BP and BCP treatments^*a*^

Gene label	Gene description	BP	BCP	Log2FC
NKI69999.1	Cation efflux system protein (CusC)	3,909	96	−5.31
NKI70000.1	Heavy metal and cation efflux efflux pump (CzcA/CusA)	7,728	189	−5.31
NKI70001.1	Membrane fusion protein/cation efflux system protein CzcB	3,424	95	−5.1
NKI69169.1	Conserved protein of unknown function	1.29E + 04	1,504	−3.04
NKI72008.1	Conserved protein of unknown function	1,382	280	−2.24
NKI68856.1	Mg(2(+)) importing P-type ATPase	151	35	−2.04
NKI72010.1	Ferric siderophore transport system, periplasmic-binding protein TonB	514	127	−1.94
NKI72009.1	Bacterioferritin-associated ferredoxin	683	173	−1.82
NKI69022.1	Vault inter-alpha-trypsin domain protein	1.02E + 05	3.05E + 04	−1.62
NKI69973.1	Conserved protein of unknown function	274	83	−1.55
NKI69168.1	D-amino acid dehydrogenase small subunit	3,117	1,051	−1.45
NKI72011.1	Biopolymer transport protein ExbB	279	100	−1.4
NKI68640.1	Conserved protein of unknown function	6,059	1,871	−1.4
NKI71576.1	Short-chain dehydrogenase family protein	160	59	−1.27
NKI69717.1	SnoaL-like polyketide cyclase family protein	301	118	−1.26
NKI71575.1	Zinc-binding dehydrogenase family protein	222	74	−1.25
NKI68435.1	C4 dicarboxylate/orotate:H(+) symporter	1,228	488	−1.22
NKI69998.1	Conserved protein of unknown function	1,029	404	−1.18
NKI68431.1	EAL domain protein	1,835	703	−1.17
NKI72568.1	Conserved protein of unknown function	4,746	1,977	−1.16
NKI71955.1	Short-chain dehydrogenase family protein	440	182	−1.12
NKI72076.1	Cell division protein FtsA	3,023	1,247	−1.12
NKI71087.1	Conserved protein of unknown function	3.15E + 04	1.30E + 04	−1.12
NKI69616.1	Ser/Thr protein phosphatase (Tat signal)	2.36E + 05	9.57E + 04	−1.07
NKI72025.1	SWIB/MDM2 domain protein	1,581	620	−1.06
NKI70430.1	Conserved protein of unknown function	744	322	−1.01
NKI70794.1	**Small subunit GMC oxidoreductase^*b*^**	1,792	818	−0.97
NKI72776.1	Dienelactone hydrolase family protein	7,460	3,388	−0.96
NKI72323.1	Carboxymuconolactone decarboxylase family protein	101	44	−0.95
NKI68787.1	Conserved protein of unknown function	253	112	−0.94
NKI70639.1	Toxic protein SymE	359	171	−0.93
NKI71346.1	Thiol:disulfide interchange protein	110	49	−0.92
NKI70934.1	Glutamate—pyruvate aminotransferase AlaC	518	252	−0.92
NKI72456.1	Conserved protein of unknown function	2,044	939	−0.91
NKI71048.1	SnoaL-like domain-containing protein	119	53	−0.9
NKI72012.1	Biopolymer transport protein exbD1	115	57	−0.9
NKI68636.1	Urea ABC transporter, urea-binding protein	71	156	0.9
NKI68636.1	Urea ABC transporter, urea-binding protein	71	156	0.9
NKI70411.1	50S ribosomal subunit protein L33	1,273	2,783	0.91
NKI71447.1	Dynamic cytoskeletal protein MreB	1,425	3,052	0.91
NKI71234.1	Cytochrome c family protein	1,613	3,509	0.91
NKI69237.1	Calcineurin-like phosphoesterase family protein	2,322	4,875	0.91
NKI71709.1	Inner membrane transport protein YajR	1,098	2,458	0.93
NKI71880.1	Phosphonoacetate hydrolase	288	593	0.95
NKI71866.1	Antimicrobial peptide resistance and lipid A acylation PagP family protein	3,355	6,782	0.95
NKI70357.1	Cystine ABC transporter membrane subunit	423	896	0.96
NKI72265.1	Bacterial regulatory s, tetR family protein	7,120	1.50E + 04	0.96
NKI71516.1	Glutaredoxin 3	1,602	3,323	0.97
NKI68990.1	Transcriptional regulator with XRE-family HTH domain	125	262	0.98
NKI69097.1	ABC-type nitrate/sulfonate/bicarbonate transport systems, periplasmic components	131	285	0.98
NKI69938.1	Conserved protein of unknown function	341	785	0.98
NKI70710.1	Serine hydroxymethyltransferase	1,157	2,405	0.99
NKI69818.1	Alternative cytochrome c oxidase polypeptide CoxM	1,150	2,484	0.99
NKI71515.1	Rhodanese-related sulfurtransferase	2,039	4,582	0.99
NKI70431.1	Protein of unknown function	758	1,606	1
NKI69522.1	Inositol 2-dehydrogenase	1,562	3,254	1
NKI70702.1	Conserved protein of unknown function	486	1,188	1.01
NKI72533.1	Efflux transporter, RND family, MFP subunit	1,058	2,334	1.01
NKI71878.1	Ferric iron ABC transporter, iron-binding protein	313	765	1.02
NKI69157.1	Multidrug efflux pump membrane subunit EmrB	563	1,308	1.03
NKI67857.1	Cytochrome c-type biogenesis protein ResA	954	2,109	1.04
NKI69437.1	Fatty acid desaturase family protein	113	274	1.05
NKI71419.1	Putative membrane protein	993	2,522	1.09
NKI69525.1	Ribose ABC transporter ATP-binding subunit	650	1,480	1.12
NKI68724.1	Major facilitator Superfamily protein	85	206	1.17
NKI69975.1	2-isopropylmalate synthase	468	1,117	1.19
NKI69163.1	Flavohemoprotein	519	1,392	1.19
NKI72565.1	Permeases of the major facilitator superfamily	519	1,285	1.2
NKI69658.1	Conserved protein of unknown function	381	1,025	1.21
NKI69155.1	Outer membrane component of tripartite multidrug resistance system	1335	3,351	1.21
NKI71310.1	Major Facilitator Superfamily protein	47	144	1.22
NKI70703.1	Conserved exported protein of unknown function	2.55E + 04	6.57E + 04	1.22
NKI68173.1	3-deoxy-7-phosphoheptulonate synthase, Phe-sensitive	695	1,785	1.27
NKI72532.1	Efflux transporter, outer membrane factor (OMF) lipo, NodT family protein	2,346	6,035	1.27
NKI68442.1	Conserved exported protein of unknown function	4,183	1.27E + 04	1.34
NKI70961.1	MarR family protein	338	1,071	1.61
NKI68441.1	Conserved exported protein of unknown function	2,948	1.05E + 04	1.7
NKI68439.1	Conserved exported protein of unknown function	2,218	8,463	1.86
NKI69384.1	Acyltransferase family protein	190	728	1.87
NKI70811.1	Conserved exported protein of unknown function	1,722	6,977	1.92
NKI70741.1	Glycine zipper family protein	6,779	2.73E + 04	1.93
NKI68440.1	Histidine kinase-, DNA gyrase B-, and HSP90-like ATPase family protein	7,226	3.14E + 04	2.06

^*a*^ Only the genes presenting an average value superior to 100 were conserved. For each, the accession number from NCBI, the number of reads in the BP and BCP treatments, and the Log2FC values are presented.

^*b*^ The GMC oxidoreductase involved in the MWe ability of strain PMB3(1) is presented in bold.

### Functional changes induced by growth in nutrient-deprived conditions

On the 2,192 and 2,104 DEGs observed, respectively, in the comparison BP vs. BBP and BCP vs. BBP, more than 1,200 appeared upregulated in the BP (medium devoid in Fe and Mg without biotite; 1,223) and BCP (complete medium without biotite; 1,210) treatments compared to BBP (medium devoid in Fe and Mg with biotite). A detailed analysis revealed that strain PMB3(1) upregulated the expression of genes related to osmoregulation, stress, metabolism/energy management, or nutrient access (Table 3).

**TABLE 3:** Top 50 list of the upregulated DEGs in absence of biotite (BP or BCP >BBP)^*a*^

Gene label	Gene description	BCP	BBP	log2FC	BP	BBP	log2FC
NKI68220.1	Saccharopine dehydrogenase NADP-binding domain	56,060	278	7.65	38,110	287	7.03
NKI69315.1	F420_oxidored domain-containing protein	47,200	443	6.7	24,720	459	5.72
NKI69316.1	Oxidoreductase (YkvO)	34,790	419	6.35	22,370	434	5.65
NKI69314.1	Transcriptional regulator family protein	13,690	231	5.84	9,777	239	5.33
NKI70564.1	Zinc chaperone 47 kDa protein	5,325	102	5.68	7,372	106	6.1
NKI70563.1	ABC transporter family protein	4,049	93	5.41	5,303	96	5.71
NKI70562.1	Zinc ABC transporter (ZnuB)	829	22	5.17	780	23	5.05
NKI68638.1	Conserved protein of unknown function	13,400	399	5.06	15,080	413	5.18
NKI68715.1	Periplasmic-binding domain protein	16,570	496	5.03	15,990	513	4.94
NKI68639.1	4-hydroxy-tetrahydrodipicolinate synthase	22,370	683	5.02	27,420	707	5.26
NKI71138.1	Methionine aminopeptidase	20,180	698	4.82	16,680	723	4.5
NKI69617.1	Cytochrome C oxidase subunit II	69,310	2,510	4.75	155,400	2599	5.84
NKI72265.1	Bacterial regulatory, tetR family protein	17,910	686	4.68	8,817	710	3.59
NKI69615.1	RNA polymerase sigma factor	21,870	847	4.64	37,150	877	5.34
NKI71137.1	Conserved protein of unknown function	5,320	215	4.59	4,766	222	4.38
NKI72126.1	D-galactonate regulator, IclR family	13,630	560	4.58	7,167	579	3.6
NKI69614.1	Zinc-finger family protein	20,250	864	4.51	30,250	894	5.03
NKI67923.1	Aminotransferase class-V family protein	39,400	1,707	4.5	49,810	1,768	4.79
NKI69616.1	Ser/Thr protein phosphatase (Tat signal)	114,700	5,204	4.42	293,100	5,388	5.72
NKI71871.1	Conserved protein of unknown function	1,655	72	4.4	1,750	75	4.23
NKI68640.1	Conserved protein of unknown function	2,235	110	4.29	7,524	114	5.98
NKI72264.1	Short-chain dehydrogenase family protein	19,730	990	4.29	17,330	1,024	4.01
NKI69743.1	Bacterial regulatory, tetR family protein	5,493	284	4.25	3,066	294	3.34
NKI69267.1	Outer membrane autotransporter barrel domain protein	4,852	270	4.13	3,792	280	3.72
NKI68064.1	Conserved protein of unknown function	7,122	393	4.11	9,273	407	4.46
NKI69744.1	Glutathione S-transferase GstA	3,670	221	4.03	2,662	228	3.52
NKI70005.1	Bacterial regulatory, tetR family protein	1,698	105	3.98	2,858	109	4.67
NKI72458.1	Bacterial regulatory helix-turn-helix, lysR family protein	5,504	360	3.92	4,717	373	3.63
NKI70909.1	Outer membrane insertion C-terminal signal domain protein	5,475	354	3.91	4,241	367	3.49
NKI70003.1	Putative membrane protein	2,395	169	3.8	2,376	175	3.74
NKI72395.1	Xanthine permease	2,188	155	3.8	2,656	160	4.03
NKI70004.1	Thioesterase-like superfamily protein	2,653	190	3.77	3,170	196	3.99
NKI72263.1	Zinc-binding dehydrogenase family protein	14,560	1,083	3.73	11,720	1121	3.3
NKI72357.1	Resolvase	51,780	3,652	3.71	29,630	3781	2.92
NKI69818.1	Alternative cytochrome c oxidase polypeptide (CoxM)	2,974	223	3.7	1,425	231	2.6
NKI71828.1	1-pyrroline-4-hydroxy-2-carboxylate deaminase	1,996	153	3.68	2,059	158	3.66
NKI71559.1	Phenylalanine-4-hydroxylase	42,310	3,326	3.65	33,880	3444	3.25
NKI70973.1	Anthranilate 1,2-dioxygenase small subunit	127	9.95	3.61	169	10	3.9
NKI69835.1	Sigma-fimbriae tip adhesin	715	57	3.58	1,266	59	4.35
NKI67924.1	DNA-binding transcriptional activator (DecR)	3,949	324	3.57	2,685	335	2.94
NKI70561.1	Periplasmic solute-binding family protein	1,045	83	3.56	941	86	3.35
NKI69831.1	Stomatin-family membrane protease subunit aq_911	6,528	554	3.52	8,350	573	3.82
NKI70522.1	DNA-binding transcriptional repressor (BetI)	4,845	412	3.51	4,959	427	3.5
NKI71786.1	Fasciclin domain protein	4,286	370	3.51	6,557	384	4.06
NKI72393.1	Cytochrome c family protein	7,048	625	3.47	6,349	648	3.25
NKI70908.1	Bacterial regulatory, tetR family protein	15,740	1,409	3.45	12,460	1,459	3.05
NKI68885.1	Efflux transporter	5,386	525	3.32	2,412	544	2.04

^*a*^ For each, the accession number from NCBI, the number of reads in the BP or BBP and BCP treatments, and the Log2FC values are presented.

With respect to osmoregulation and stress management, the most regulated genes were assigned to the saccharopine pathway (saccharopine dehydrogenase (NKI68220.1)(first upregulated gene/Log2FC:7.03) and related genes (NKI68639.1 = 4-hydroxy-tetrahydrodipicolinate synthase, Log2FC:5.26 ; 1-pyrroline-4-hydroxy-2-carboxylate deaminase, Log2FC:3.66), hydantoin (permease, NKI68782.1 = 2.21 and racemase, NKI68783.1 = 0.95), and betaine (betaine aldehyde dehydrogenase (NKI70521.1, Log2FC = 1.91; glycine/betaine ABC transporter (NKI72230.1; OsmF), Log2FC = 0.83; 5-oxoprolinase subunit A,B,C (enzyme involved in the conversion of proline to glutamate, NKI71381.1 [Log2FC :0.86 ; A] to NKI71383.1 [Log2FC :1.66 ; B]). Many transporters associated with solute export appeared also upregulated (ABC transporters, NKI70563.1 [Log2FC :5.71]; NKI72150.1 [Log2FC :2.18], NKI72151.1 [Log2FC :1.85]) or to cation transport as homologues of the K(+) transporting P-type ATPase (Kdp) (KdpA,B,C, F: NKI70317.1 [Log2FC :2.23] to NKI70320.1 [Log2FC :3]) or zinc/manganese ABC transporter (ZnuB) (Log2FC :5.05; NKI70562.1). Considering stress adaptation, several putative methionine sulfoxide reductases (MsrA/B like) appeared differentially regulated (e.g., NKI70018.1 [MsrA, Log2FC :1.94]; NKI69912.1 [MsrB, Log2FC :2.42] or methionine-sulfoxide reductase subunit YedZ1 [NKI72642.1 and NKI72643.1]).

Modification of the metabolism was also visible at different levels with DEGs encoding enzymes in charge of phosphorus recovery from phosphorylated molecules, energy management, and conservation through various cytochrome c oxidases and other cellular processes. A gene coding for a Ser/Thr protein phosphatase (NKI69616.1), an enzyme in charge of removing the serine- or threonine-bound phosphate group from a wide range of phosphoproteins, was in the top 10 of the most upregulated gene (Log2FC:5.72), and several other phosphatases were detected such as a phosphatase NudJ (NKI72618.1 [Log2FC :1.28]), a phosphatase 2C (NKI69689.1 [Log2FC :1.27]) or trehalose-6-P phosphatase (NKI70021.1 [Log2FC :1.53]). More than 15 genes associated with cytochrome c oxidase or cytochrome oxidase biogenesis appeared upregulated in our data set. Cytochrome c oxidases involved in the respiratory chain appeared upregulated with Log2FC ranging from 5.84 for NKI69617.1 (identified as coxM and part of a cluster formed by coxPOQM), 3.25 for NKI72393.1 (cytochrome c oxidase) to 1.66 for cytochrome bd-I ubiquinol oxidase (cyd, NKI68732.1) and 0.93 for a cytochrome oxidase biogenesis (surf, NKI71798.1). Apart from them, genes associated with oxalate degradation appeared also upregulated (NKI69445.1, formyl-CoA transferase NAD(P)-binding oxalate/Log2FC:1.17; NKI69446.1, oxalyl-CoA oxalate decarboxylase/Log2FC:1.4 and NKI69451.1, putative oxalate/formate antiporter/Log2FC:1.93).

With respect to nutrient access, several genes related to iron access and homeostasis appeared upregulated. Genes associated with mobilization of iron appeared upregulated as genes of the ftr system (ftrA,B,C) (NKI69657.1 (Log2FC:2.61); NKI69656.1 (Log2FC:2.57); NKI69655.1 (Log2FC:2.15)) or associated with the production of malleobactin (but expressed at a relatively low level) such as the NRPS synthetase (MbaB, NKI69296.1/Log2FC: 0.91), siderophore transporter (MbaI, NKI69290.1 [Log2FC:0.95]), and a sigma factor (MbaF, NKI69287.1 (Log2FC:1.47)). Noticeably, several genes associated with the transport of ferric siderophore were also detected, such as TonB and ExbB/ExbD transporters (NKI68680.1 [Log2FC:4.14]) or (NKI72011.1/NKI72012.1 [Log2FC:2.08/1.89]). In addition, genes associated with mobilization of intracellular iron using bacterioferritin ferredoxins (NKI69654.1 [log2FC:1.47]; NKI70697.1 [log2FC:1.1]; NKI72009.1 [log2FC:2.77]) or phosphorus using Ser/Thr protein phosphatase (NKI69616.1 [log2FC:5.72]), Patatin-like phospholipase (NKI69822.1 [log2FC:1.74]) were also detected.

### Functional changes induced in the presence of biotite

The comparisons done between the treatment where biotite was introduced and the other treatments (BBP vs. BP or BBP vs. BCP) revealed a very similar set of genes regulated (Table 4 for top upregulated in BBP or BCP), showing that the main physiological adjustments done by PMB3(1) corresponded to the presence/absence of biotite. More than 80% of DEGs were common in the two comparisons (i.e., BP vs. BBP or BCP vs. BBP), meaning that BP and BCP were very similar. The differences mostly corresponded to genes with low expression levels that were not in the top regulated genes. For this reason, we mainly used the comparison BCP vs BBP. A detailed analysis revealed that strain PMB3(1) upregulated the expression of genes related to adherence to the surface, motility, cation transport, and chemotaxis.

**TABLE 4:** Top 50 list of the up-regulated DEGs in presence of biotite (BBP >to BP or BCP)^*a*^

Gene label	Gene description	BCP	BBP	log2FC	BP	BBP	log2FC
NKI72714.1	Autotransporter adhesin protein	44	18,910	−8.75	51	19,600	−8.59
NKI72715.1	Autotransporter adhesin protein	74	15,710	−7.7	83	16,300	−7.6
NKI69906.1	Fimbrial family protein	49	7,908	−7.3	26	8,194	−8.3
NKI69999.1	Cation efflux system protein (CusC)	115	12,270	−6.71	4,844	12,700	−1.37
NKI68856.1	Mg(2(+)) importing P-type ATPase	42	3,556	−6.42	188	3,682	−4.27
NKI70000.1	Heavy metal/cation efflux pump (CzcA/CusA)	227	18,740	−6.33	9,578	19,400	−0.99
NKI72712.1	Autotransporter adhesin protein	23	1,468	−5.94	33	1,520	−5.49
NKI69431.1	Methyltransferase domain protein	341	17,350	−5.65	241	18,000	−6.2
NKI69904.1	Outer membrane usher protein LpfC	18	896	−5.56	30	927	−4.93
NKI72599.1	MltA-interacting MipA family protein	33	1,516	−5.48	39	1,571	−5.32
NKI71256.1	Putative membrane protein	17	831	−5.45	29	860	−4.83
NKI69432.1	Taurine catabolism dioxygenase (TauD)	216	9,456	−5.44	178	9,792	−5.76
NKI69905.1	Fimbrial chaperone (LpfB)	8.38	308	−5.12	8.13	319	−5.23
NKI69438.1	Diaminobutyrate-2-oxoglutarate aminotransferase	312	10,550	−5.07	198	10,900	−5.76
NKI69429.1	Acyl-CoA dehydrogenase, C-terminal domain protein	624	20,770	−5.03	480	21,500	−5.47
NKI72708.1	Conserved protein of unknown function	12	428	−4.99	12	443	−5.14
NKI69433.1	ABC transporter family protein	244	7,176	−4.86	142	7,431	−5.69
NKI69430.1	Citrate synthase family protein	457	12,760	−4.79	320	13,200	−5.34
NKI69426.1	Diaminopimelate decarboxylase	250	6,987	−4.76	300	7,238	−4.56
NKI69428.1	3-Oxoacyl-[acyl-carrier-(ACP)] synthase III C terminal family protein	518	14,130	−4.75	389	14,600	−5.21
NKI71118.1	tRNA synthetases class I family protein	1,345	36,690	−4.75	1,582	38,000	−4.57
NKI68090.1	Conserved protein of unknown function	41	1,093	−4.73	39	1,133	−4.81
NKI69427.1	AMP-binding enzyme family protein	445	11,940	−4.72	422	12,400	−4.86
NKI68636.1	Urea ABC transporter, urea-binding protein	187	4,832	−4.68	88	5,004	−5.82
NKI69436.1	FAD-linked oxidase, C-terminal domain protein	266	6,809	−4.66	181	7,053	−5.24
NKI69133.1	DNA-binding protein HU-beta	292	7,251	−4.61	264	7,505	−4.78
NKI71119.1	Aspartate aminotransferase	968	23,320	−4.57	933	24,200	−4.68
NKI71117.1	Cupin domain protein	695	16,500	−4.54	792	17,100	−4.4
NKI69425.1	3-Oxoacyl-[acyl-carrier-(ACP)] synthase III family protein	414	9,544	−4.49	483	9,887	−4.33
NKI68633.1	Urea ABC transporter. ATPase protein (UrtD)	49	1,116	−4.45	39	1,155	−4.88
NKI69434.1	ABC-2 type transporter family protein	209	4,199	−4.32	115	4,349	−5.2
NKI69439.1	Alpha-ketoglutaric semialdehyde dehydrogenase 2	491	9,910	−4.3	314	10,300	−5.01
NKI71054.1	Alpha/beta hydrolase fold family protein	4.11	99	−4.3	11	102	−3.16
NKI69437.1	Fatty acid desaturase	329	6,426	−4.25	140	6,656	−5.56
NKI71190.1	ATP synthase F1 complex subunit epsilon	255	4,887	−4.23	313	5,058	−3.99
NKI68635.1	Urea ABC transporter, ATPase protein (UrtD)	51	969	−4.2	35	1,004	−4.8
NKI72184.1	Conserved protein of unknown function	48	898	−4.17	54	930	−4.07
NKI71116.1	2OG-Fe(II) oxygenase superfamily protein	1,500	27,280	−4.16	1,366	28,200	−4.34
NKI68632.1	Branched chain amino acid/phenylalanine ABC transporter ATP-binding subunit (LivF)	41	738	−4.14	40	765	−4.26
NKI69435.1	Putative membrane protein	308	5,517	−4.14	185	5,714	−4.92
NKI71543.1	Gram-negative porin	190	3,388	−4.13	241	3,509	−3.85
NKI72191.1	Bacterial type II and III secretion system	32	596	−4.12	51	617	−3.58
NKI68634.1	Urea ABC transporter, permease protein UrtC	38	672	−4.11	36	696	−4.25
NKI69421.1	Phosphopantetheine attachment site family protein	477	8,260	−4.08	638	8,556	−3.7
NKI69903.1	Fimbrial family protein	30	515	−4.08	44	533	−3.55
NKI69424.1	p-Aminobenzoate N-oxygenase (AurF)	617	10,570	−4.04	690	11,000	−3.94
NKI71147.1	Hep_Hag family protein	459	7,548	−4.02	707	7,817	−3.45
NKI70380.1	Ketol-acid reductoisomerase (NADP(+))	631	10,130	−3.99	732	10,500	−3.81
NKI67874.1	Signal transduction histidine kinase (CheA)	1,065	16,970	−3.97	1,330	17,600	c3.69
NKI70541.1	Lead, cadmium, zinc, and mercury transporting ATPase	317	4,885	−3.93	250	5,058	−4.32

^*a*^ For each, the accession number from NCBI, the number of reads in the BP or BBP and BCP treatments, and the Log2FC values are presented.

Among the most differentially upregulated genes, genes related to surface interaction and adherence (i.e*.,* adhesin autotransporter and fimbriae) were identified, suggesting an important role for strain PMB3(1) in the presence of biotite. Noticeably, this category was found in the top 10 genes of the most upregulated genes with autotransporter adhesin protein (NKI72712.1 [log2FC: −5.49]; NKI72714.1 [log2FC: −8.59]; NKI72715.1 [log2FC: −7.6]), fimbriae-related proteins (NKI69904.1 [LpfC], log2FC: −4.93); NKI69905.1 (LpfB, log2FC: −5.23); and NKI69906.1 (LpfA, log2FC: −8.3). In addition to these genes, several genes related to the pilus biogenesis/assembly and chemotactism appeared upregulated. This is the case of several genes involved in type IV pilus biogenesis, mainly organized in two cluster genes (from NKI71689.1 to NKI71692.1 [Pil N,O,P,Q; log2FC: −1.07 to −1.66]; from NKI72651.1 to NKI72659.1 [PilX,TapA,PilV,X,Y1; log2FC: −0.91 to −1.57]; and some other genes encoding PilG [log2FC:−1.7], PilH [log2FC:−2.31], PilJ [log2FC:−1.98], TadD [log2FC:−0.91], and TadE [log2FC:−1.91]). Chemotactism-related genes were also upregulated (e.g*.,* NKI69215.1 [CheW, log2FC:−2.72]; NKI69217.1 [log2FC:−2.25]; NKI69218.1 [log2FC:−2.36]; NKI67874.1 [CheA, log2FC:−3.7]).

Another important category of DEGs was related to transport and sensing (e.g., ABC transporter, secretion systems, Major Facilitator Superfamily [MFS] proteins, multidrug efflux pump, symporters). More than 52 genes assigned to ABC transporters, related to the transport of organic compounds, such as urea, glutamate/aspartate, glutathione, glucose, putrescine, methionine, ribose, or metals (iron, molybdenum), were upregulated in the presence of biotite. As an example, a genomic region in charge of urea transport was upregulated (NKI68633.1 to NKI68636.1; [UtrABCD [log2FC: −4.88 to −5.82]). Similarly, a system in charge of the recycling glycerophospholipid metabolites was up-regulated (NKI70463.1 to NKI70466.1; UgpABCE [log2FC: −1.7 to −3.77]). Noticeably, apart from these ABC transporters, other transport and secretion systems appeared up-regulated. This is the case of glucose (NKI70109.1 [log2FC:−1.66]) and ammonium transport (NKI71404.1 [AmtB], log2FC:−3.54). Several genes associated with Sec or type I, II, III, IV, and VI secretion systems appeared upregulated (log2FC: from −0.9 [for NKI71520.1, gspG, type II] to −3.1 [NKI72191.1, type II/III]). In addition, several transport or sensing systems related to magnesium (Mg^2+^ importing P-type ATPase NKI72539.1 [log2FC: −2.03] and NKI68856.1 [log2FC:−4.27]), lead/cadmium (NKI70541.1/log2FC:−4.32), or potassium (NKI71393.1 [Kup, log2FC:−1.91]; NKI72441.1 [KefA, log2FC:−0.84]) appeared upregulated. Adjustments related to iron were also visible through upregulation of genes related to FecR (NKI68785.1 [log2FC:−1]), Fe3 +import (fbpC) (NKI71877.1 [log2FC:−1.15]), and iron storage (bfr bacterioferritin, NKI72013.1 [log2FC:−1.96]).

At last, several genes related to metabolism were upregulated as those involved in glucose metabolism, mannose conversion, and organic acid production. Among them, we identified the system in charge of glucose conversion to gluconate (i.e., GMC oxidoreductase large and small subunits, NKI70794.1/NKI70795.1 [log2FC:−1.09/1.02]), known to confer its effectiveness at weathering minerals to strain PMB3(1) through an acidification-driven mechanism. Several genes associated with mannose (e.g., Alpha-1,2-mannosidase, mannose isomerase, dehydratase) and trehalose metabolism (e.g., trehalase), malate (malate synthase) and citrate (citrate synthase and citrate/H + symporter) production/transport were also upregulated as well as several cytochrome C oxidases (e.g., NKI71793.1 to NKI71796.1 [ctaD/G], log2FC: −1.78 to −2.52; NKI72210.1/NKI72211.1 [cytoC], log2FC:−1.32 to −1.43).

## DISCUSSION

The conserved ability of *Collimonas* to weather minerals (29, 35), their occurrence in nutrient-poor environments, and particularly on mineral particles (24, 31), led us to hypothesize that representatives of this genus are well adapted to nutrient depletion and to mobilize nutrients from recalcitrant origin (i.e*.*, minerals), probably through the regulation of various physiological processes. In previous studies, we identified the main mechanisms used by different strains of *Collimonas* (i.e*.,* acidification- and chelation-driven MWe mechanisms) (41) to weather minerals and functionally characterized the genes associated (i.e., GMC oxidoreductase and malleobactin) (36, 37) for the strain *Collimonas pratensis* PMB3(1). A deep analysis of the genome of strain PMB3(1) highlighted that outside of the NRPS system encoding malleobactin, this strain possesses a complex set of genes homologous to iron mobilization and perception systems such as fecIR and fur (fecIR, [NKI68784.1–NKI68785.1]; fur, NKI71994.1), acquisition (i.e., the *ftrABCD* system, [NKI69657.1-NKI69654.1]; *hmu* system, [NKI72429.1, NKI72430.1]) and storage (i.e., bacterioferritin [NKI72009.1-NKI72013.1]). These genes may enhance the effectiveness of weathering of strain PMB3(1), alongside other genes whose functional roles remain either uncharacterized or associated with unrelated processes (i.e., motility, cation transport).

In this study, we considered an early stage of interaction between biotite and bacteria to maintain the maximum nutrient depletion and maximize the potential regulations employed by strain PMB3(1) to adapt to these depleted conditions and weather minerals. While the short-term incubation time considered can be viewed as a limitation for processes occurring at longer terms, it clearly permitted highlighting significant changes of pH and nutrient concentrations of the solution, meaning that 20 h was enough for strain PMB3(1) to promote biotite dissolution. The effectiveness at weathering minerals of bacteria has typically been considered after longer incubation times (i.e., 48 h to several days), where the availability of initial carbon substrate can be questioned (26, 38, 42–45). After 20 h, bacteria still have available glucose for their metabolism, that is not the case after incubations longer than 48 h, where the initial substrate has been converted into byproducts (36, 46). Another important consideration during this early stage of interaction is the exposure of the bacteria to nutrient-depleted conditions, where they must mobilize both intracellular and extracellular resources, circumstances that may not apply during longer incubation periods. In our study, the changes occurring in the solution chemistry evidenced a strong acidification, as the pH goes from 6.3 to ca. 3, highlighting that strain PMB3(1) employed an acidification-driven MWe mechanism to weather biotite. Noticeably, this acidification appeared stronger in the absence of biotite (i.e., pH_BP_ = 3.01 vs. pH_BBP_ = 3.26) as well as the concentration of gluconic acid (i.e., without biotite [BP = BCP] = 1,200 mg.L^−1^ vs. with biotite [BBP] = 711 mg.L^−1^). A higher production of gluconic acid in nutrient-depleted conditions was already evidenced for different bacterial strains such as *Caballeronia mineralivorans* PML1(12), *Paenibacillus sonchi* SBR5, and *Pseudomonas putida* KT2440 (47–49). This change has been explained as an adjustment of the bacterial metabolism according to nutrient depletion and differential secretion of gluconic acid in the extracellular environment (49, 50). In replete conditions, glucose is converted into energy and biomass, while in depleted conditions, especially of iron, glucose is employed for the production of siderophore and in the direct oxidative pathway for the production of gluconic acid, and poorly for bacterial growth (49, 51). Another explanation is related to the buffer capacity of biotite during its dissolution, which may consume the protons produced by bacteria. In our experimental conditions, no production of malleobactin has been observed as stated by siderophore assay or based on the transcriptomic/proteomic data, while the concentrations of iron and aluminum in solution were far lower than the demonstrated inhibiting thresholds for strain PMB3(1) (i.e., [Fe]>0.8 mg.L^−1^ and [Al]> 1 mg.L^−1^ (37)). Noticeably, a similar observation has been made for strain *Caballeronia mineralivorans* PML1(12) (46, 52). The absence of detectable production of siderophore, a molecule with an important metabolic cost, may be explained by the low growth associated with the acidification-driven MWe mechanism employed by these effective MWe strains in nutrient-poor conditions and the early stage considered (i.e., 20 h) or by alternative uncharacterized regulatory mechanisms (53).

One hypothesis tested here was that nutrient availability is a key factor determining the expression of various functions in strain PMB3(1). In this study, we considered the effect of the depletion of two nutrients (i.e., Fe and Mg) that are found in biotite and other minerals, typically limiting in forest soils, and which are released from minerals during the MWe process, may impact the physiology, competitiveness, and effectiveness at weathering of strain PMB3(1). The questioning on the relative impact of nutrient depletion (i.e., P, Fe, or Mg) has already been investigated on a few other model bacterial strains in relation to their response to nutrient availability and/or their ability to weather and transform minerals (e.g., biotite, basalt, serpentine, and hydroxyapatite) (43–45, 47–49, 54, 55). In terms of solution chemistry, no change was observed (i.e., absence of detectable siderophore activity, same concentrations of gluconic acid and same concentrations of Ca, Al, Mn, Si) between the BP (medium devoid of Fe and Mg) and BCP (complete medium) treatments considered in our study, except for the concentrations of Mg and Fe, with an expected greater concentration in the complete medium (BCP) treatment. At the transcriptomic and proteomic levels, only a few genes/proteins differentially regulated have been detected (i.e*.*, 196 DEGs and 29 DAPs). These results fit with those of Bryce et al. (54), which also showed a limited effect of Fe and Mg depletion on the model strain *Cupriavidus metallidurans* CH34 at the proteome level, but at the same time, a better growth in the amended condition. No improvement in the growth of strain *Collimonas pratensis* PMB3(1) was observed in our experimental conditions in the complete medium (BCP) treatment. A detailed analysis revealed that half of the DEGs were upregulated in the deprived treatment. Most of these genes were related to cation transporters (e.g., CusABC; Mg2 +importing P-type ATPase), siderophore receptors (e.g., TonB, ExbB), and iron storage management (e.g., bacterioferritin). By contrast, this analysis highlighted that among the DEGs upregulated in the BP treatment, a higher expression was observed for the GMC oxidoreductase (NKI70794.1 [log2FC: −0.97]), suggesting that the expression of this enzyme, known to confer the acidification-driven MWe ability to strain PMB3(1), is regulated according to the availability of Fe and Mg.

The comparison done between the nutrient-depleted condition and the condition where biotite was introduced permitted the investigation of the regulations occurring in nutrient-poor conditions (i.e., higher expression in the BP treatment). This treatment can be considered as the step experienced by bacteria in their environment just before interaction with minerals and when nutrient availability is still low (56–58). Noticeably, the comparisons done between the treatments with (BBP) and without biotite (BP and BCP) amendment clearly evidenced that the BP and BCP treatments were very similar in terms of DEGs, meaning that though Mg and Fe were added, the BHm medium remains a nutrient-poor medium compared to its version amended in presence of biotite (BBP). The ability to survive and to be active in nutrient-poor conditions represents an ecological advantage for bacteria, but these conditions also represent important sources of stress due to the changes in pH, nutrient availability, oxidoreduction reactions, and production of reactive oxygen species (56, 57). Our analyses revealed an upregulation of several genes associated with mechanisms of adaptation based on osmoregulation, sensing, and the recovery of intracellular and extracellular nutrients. Osmoregulation represented the most upregulated category of genes of strain PMB3(1) in the BP and BCP treatments compared to the treatment where biotite was introduced (BBP), with DEGs associated with the saccharopine pathway, the production and transport of hydantoin, betaine, glycine, glutamate, or trehalose. These metabolites correspond to solutes (or osmolytes) produced by bacteria in conditions where the intracellular and extracellular environments strongly differ (59, 60). They are acting as osmoprotective compounds together with the stimulation of K uptake, as evidenced in our study with the upregulation of the Kdp system. The differential expression evidenced in our study shows how strain PMB3(1) adapts to low nutrient availability to maintain its internal osmotic pressure and metabolic activities. Increased concentrations of proline, trehalose, and glycine betaine were also reported for the strain *Paenibacillus sonchi* when it was growing under low P availability compared to high P availability (47). The potential direct or indirect roles of these metabolites in the MWe process remain to be demonstrated. Outside of this first adaptation, bacteria are probably experiencing multi-nutrient deficiencies and stresses (56–58). One expected response of bacteria living in such conditions would be the optimization of their ability to mobilize nutrients. In this sense, we observed that strain PMB3(1) activated processes involved in the mobilization of intracellular P and Fe through the upregulation of genes involved in the production of enzymes to recover P from P-carrying molecules (e.g., phosphoesters, phospholipids) and Fe from bacterioferritin, and in the mobilization of extracellular Fe and K through the upregulation of different transporters: (i) the FtrABCD system for iron (61) and (ii) the Kdp system for K (62). The FtrABCD transporter system has been shown to function better under acidic conditions, which correspond to the increased solubility of iron at low pH (61, 63). This aligns with the condition observed in our study, as well as those found in the soil from which strain PMB3(1) originates. Interestingly, the activation of nutrient mobilization mechanisms from intracellular and extracellular origins was also observed for *Cupriavidus metallidurans* CH34 when incubated in depleted conditions in the absence of basalt (54). Noticeably, the GMC oxidoreductase of strain PMB3(1), the enzyme in charge of the conversion of glucose to protons and gluconic acid, did not appear upregulated in the depleted treatment compared to the treatment where biotite was introduced (GMC expression: BP <BBP), while a higher concentration of gluconic acid was detected. The question was here to determine whether this change corresponded to (i) a regulation of the GMC oxidoreductase at another level, (ii) a consumption of gluconic acid by the biotite surfaces, (iii) a metabolic adjustment, or (iv) a by-effect of the biotite dissolution. A detailed analysis of the RNAseq data revealed relatively similar expression of the genes encoding the small, large, and cytochrome subunits of the GMC oxidoreductase of strain PMB3(1) in all the treatments, with the BCP treatment characterized by the lower expression compared to BBP. Noticeably, the genes in charge of the export (i.e., TatA and TaB) of the small subunit of the GMC oxidoreductase (which contains a TAT signal) in the periplasm, and of the maturation of the cytochrome subunit of the GMC oxidoreductase (i.e., ResA) were also upregulated in the absence of biotite (BP or BCP >BBP). All these genes have previously been highlighted by random mutagenesis on the strain PMB3(1) as essential for the acidification-driven MWe mechanism (36).

Outside of the microbial response to nutrient depletion, an important point is to elucidate how microorganisms interact and weather minerals. Minerals and rocks have long been regarded as merely inert and stable physical surfaces, with no or poor reactivity and no significant influence on microbial physiology. As a consequence, limited information is available on aerobic systems, while this field has been deeply developed for anoxic systems. This is a paradoxical situation as minerals and rocks have been the first support of life before the development of the soil, and they remain the main constituents of soil. At the ecological level, minerals and rocks are essential reservoirs of nutritive elements for the long-lasting functioning of forest ecosystems. They represent reactive interfaces that change the pH locally and/or provide nutrients (64). They also represent a particular habitat where microorganisms can be protected from predators and environmental conditions in cracks and holes. Indeed, it is more and more clear that complex microbial communities are inhabiting mineral surfaces and that this colonization is determined by the physicochemical properties of the minerals and the functional abilities of these microorganisms (56, 64, 65). At the organism level, bacterial/mineral interactions and weathering seem to depend on the mineral properties, the nutritive requirements of the microorganisms, and on the development of a complex suite of microbial processes (43, 52, 58). At the molecular level, our understanding remains very limited, highlighting the need for straightforward experiments to uncover the mechanisms involved (43–45, 48, 54, 55, 66). Deciphering the mechanisms employed at these different scales is essential both in terms of knowledge regarding the functioning of nutrient-poor environments and the potential feedback effects to consider, but also in terms of management to maintain in the long term the productivity and the biodiversity of these ecosystems. In our experimental conditions, the first class of mechanisms evidenced corresponded to nutrient and surface sensing, which presented the most upregulated genes. Indeed, genes expected to play a role in surface adhesion, chemotactism (CheA,W), fimbriae (lpfABC), and pili (PilHGJ, TadDE) production and activation were detected. Interestingly, upregulation of genes related to the functions listed above was also reported for other model bacterial strains (*Caballeronia, Priestia, Pseudomonas, Bacillus*) in the presence of different minerals (biotite, feldspar, serpentine) (45, 48, 55), suggesting that part of these responses may be conserved. At the same time, no biofilm formation was observed in our experimental conditions, most of the cells remaining as planktonic in the solution. We already pointed out this absence of biofilm development for various bacterial strains using the same BHm culture medium. This absence of biofilm development may be due to the stage observed that was too early to observe a biofilm, to the culture conditions (i.e*.,* chemical composition, carbon source, shaking, and incubation time), and to the final acidic pH of the solution. Regardless of these hypotheses, our data revealed the activation of surface detection mechanisms but not of mechanisms related to biofilm formation. Noticeably, the motility of strain PMB3(1) on the surface of agar culture medium has already been shown to depend on twitching and on the type IV pili (T4P) system (67). The T4P system (PilC, D, M, etc.) has been shown to permit the adhesion on different surfaces (e.g., chitin) (68) and at the same time to condition transition from planktonic to surface-adapted state (69), suggesting a role in the interaction with mineral particles and possibly their colonization. The second class of mechanisms identified corresponded to the nutrient transport (MFS, ABC transporters) and more especially to the import of Mg, K, Fe, or NH_4_^+^. For iron, an upregulation of genes involved in the storage process was observed in the BBP treatment, differing from the depleted condition (BP or BCP), where iron was mobilized from intracellular reserve (i.e., bacterioferritin). The last class of mechanisms identified corresponded to carbon metabolism. Outside of the upregulation of the GMC oxidoreductase involved in the conversion of glucose to gluconic acid and protons (36), a global increase in the expression of genes related to mannose, trehalose, or malate metabolism was observed. Upregulation of the carbon metabolic pathway and particularly of various oxidoreductases has been proposed as the main mechanism by which bacteria weather minerals (43, 66). The question remains whether this upregulation results from the release of nutrients from minerals, which better sustain the bacteria, or from the activation of specific processes dedicated to the promotion of mineral weathering.

### Conclusion

The combination of geochemical and omics approaches, along with the integration of diverse biological responses and the chemical changes observed in solution chemistry, provided deeper and novel insights into the regulatory mechanisms at play during the early stages of interaction between the strain *Collimonas pratensis* PMB3(1) and biotite. The chemical analyses done on the solution clearly evidenced the relative impact of strain PMB3(1) on biotite dissolution as well as the important role of glucose and its conversion to protons and gluconic acid. Noticeably, the integration of proteomic and transcriptomic data offers a robust perspective on these regulatory processes. While proteomics provides valuable information directly linked to the phenotype (as proteins are key to metabolism), it can sometimes be less comprehensive than transcriptomics due to challenges in protein detection and quantification. The observed regulations clearly illustrate how the nutritional needs of this strain activated a broad range of functions related to osmoregulation, nutrient transport, and nutrient mobilization from both intracellular and extracellular sources. At the same time, the responses elicited in the presence of biotite extend beyond simple reactions to magnesium and iron depletion, as applied in this study. This suggests that mineral/bacterial interactions represent a promising field of research that warrants further investigation in geomicrobiology, but also in different fields related to soil formation, biogeochemical cycling, or material conservation. An important step would be to consider over a longer incubation period the physiological responses of effective MWe bacteria, as well as how the mechanisms employed are regulated or even change from *in vitro* and *in situ* experiments. These new findings will be necessary to permit environmental engineering and biotechnological applications based on controlled plant growth promotion or on selective metal extraction using MWe bacteria.

Interestingly, while research on microorganisms/minerals interactions and weathering remains limited, many of the functions and regulatory mechanisms identified here are consistent with findings on other model bacterial or fungal strains proficient in weathering, suggesting the existence of common underlying mechanisms, even if the specific genes or type of enzymes involved may vary. For example, the upregulation of nutrient transporters (e.g., potassium transporters), oxidoreduction pathways, and acid production (e.g., oxalate) has also been observed in fungi (70, 71), indicating that similar global strategies may be employed by diverse organisms. Expanding the number of studies investigating the molecular mechanisms fungi use to weather minerals would provide valuable insights. Since *Collimonas* are typically associated with fungi in nutrient-poor environments, future research could also focus on whether and how interactions between *Collimonas* and fungi contribute significantly to the mineral weathering process in such environments. This could be done using dual transcriptomics on microcosm systems or even with tree seedlings.

## MATERIALS AND METHODS

### Bacterial strain and culture media descriptions

Strain PMB3(1) was used in our study as a model organism due to its effectiveness at weathering minerals (35, 38, 39). This strain is currently identified as *Collimonas pratensis* (72). Representatives of this genus are particularly enriched in the mycorrhizosphere (32) or in the mineralosphere (31, 73) and to be negatively impacted by an increase of Mg and K availability (28). Strain PMB3(1) was grown at 25°C on solid and liquid low salt LB medium (LBm). The LBm composition is as follows (g.L^−1^): NaCl, 1; Tryptone, 10 and 5, Yeast extract. For the microcosm experiment described below, the Bushnell-Haas (BHm) medium was used. The BHm composition is as follows (g.L^−1^): KCl, 0.020; NaH_2_PO_4_,2H_2_O, 0.080; Na_2_HPO_4_,2H_2_O, 0.090; (NH_4_)_2_,SO_4_, 0.065; 0.2 MgSO_4_,7H_2_0, 0.2; KNO_3_, 0.100; CaCl_2_, 0.020, and 0.05 FeCl_3_,6H_2_O. To create a nutritive depletion complementary to the nutrients carried by the mineral used (i.e., the biotite), a modified version of this BHm medium devoid of Mg and Fe was also used. This depletion was justified by five main reasons: (i) Fe and Mg are usually found in low concentrations in acidic and nutrient-poor forest soils, (ii) Fe and Mg are important nutrients for bacterial physiology, (iii) Fe and Mg are found in minerals and rocks, (iv) Fe and Mg can be removed from the culture medium without strong impact on bacteria; and (v) by depleting the culture medium of these elements, the only source of Fe and Mg is the biotite introduced, facilitating the evaluation of biotite dissolution. In both conditions, the pH of the medium was adjusted to 6.5 and glucose was used as the sole carbon source (2 g.L^−1^). This substrate was used as it confers to strain PMB3(1) the higher effectiveness at weathering compared to the other carbon sources tested (35).

### Mineral description

Biotite was used in this study because it is a mineral frequently found in acidic soils. The soil, from which the strain PMB3(1) was isolated, is developed on granite that contains biotite. The batch used comes from Bancroft (Canada). Its chemical composition is (g.kg^−1^): 410.1 SiO_2_, 109.0 Al_2_O_3_, 22.1 Fe_2_O_3_, 100.5 FeO, 2.7 MnO, 189.0 MgO, 4.1 Na_2_O, 94.6 K_2_O, 22.8 TiO_2_, 44.2 F, and 0.8 Zn (40). Its structural formula is (Si_3_Al_1_) (Fe^3+^_0.12_ Fe^2+^_0.61_ Mg_2.06_ Mn_0.02_ Ti_0.13_) and K_0.88_ Na_0.06_ O10 (OH_0.98_F_1.02_). Size selection was performed to conserve only the 200–400 µm particles. The release of weak amounts of nutrients is known to occur when minerals are introduced in aqueous solution (i.e*.,* passive dissolution) (74).

### Microcosm experiment

Hundred-mL capped Erlenmeyer flasks filled with 45 mL of sterile BHm medium, devoid or not of Fe and Mg, have been used. In these microcosms, 2 g of autoclaved biotite particles (size: 200–400 μm) has been introduced depending on the treatment considered. All glassware was washed with 3% HCl and rinsed with deionized water. For each microcosm, a volume of 5 mL of sterile water or inoculum was added, giving a total volume of 50 mL. For each treatment, four flasks were prepared. Inoculum was prepared by inoculating a single colony in 50 mL of LBm medium. Briefly, after 48 h time incubation at 25°C, the culture was centrifuged and washed three times (8,000 *× g* 15 min at 4°C) in sterile water and suspended in 5 mL of sterile water to obtain a calibrated suspension at optical density (OD) 595 nm corresponding to 5. 10^9^ cell.mL^−1^. The final concentration of cells was about ca 5. 10^8^ cell.mL^−1^ in the culture medium in each inoculated flask. Our experimental design (Fig. S1) was determined as follows: (i) **B**, **B**Hm medium devoid of Fe and Mg; (ii) **BB**, **B**Hm medium devoid of Fe and Mg with **B**iotite; (iii) **BC**, **B**Hm medium **C**omplete (containing Fe and Mg); (iv) **BP**, **B**Hm medium devoid of Fe and Mg inoculated with the strain **P**MB3(1); (v) **BBP**, **B**Hm medium devoid of Fe and Mg with **B**iotite and inoculated with the strain **P**MB3(1); (vi) **BCP**, **B**Hm medium **C**omplete (containing Fe and Mg) with the strain **P**MB3(1). Our experimental design permitted the comparisons between (i) BP (depleted medium without biotite +bacteria) vs BBP (depleted medium with biotite +bacteria); (ii) BP (depleted medium without biotite +bacteria) vs BCP (complete BHm medium without biotite +bacteria); (iii) BBP (depleted medium with biotite +bacteria) vs BCP (complete BHm medium without biotite +bacteria); and (iv) abiotic (B, BB, BC) and biotic (BP, BBP, BCP) treatments for the solution analyses. The abiotic controls permitted determining the level of passive dissolution and the impact of strain PMB3(1) on the solution chemistry. No treatment BCP where biotite was introduced was considered, as strain PMB3(1) is not effective at weathering in such conditions. Microcosms were incubated at 25°C with agitation at 140 rpm in an orbital Minitron shaker (Infors). The incubation was stopped after 20 h, as it corresponds to the shorter incubation time with which the amount of Fe released in solution can be measured accurately, and when glucose is still available for bacterial metabolism.

### Chemical and biological analyses

For each replicate, 1 mL of culture was collected to determine the cell count through a serial dilution/plating on LBm agar medium. Colony-forming units (cfu) were determined after 3 days of culture at 25°C. To perform solution analyses, a volume of 30 mL of culture was centrifuged (9,000 × *g* during 20 min) and the resulting supernatant was filtered at 0.22 µm (GHP Acrodisc 25 mm syringe filter; Pall). This filtered supernatant was used to determine (i) the chemistry of the solution and (ii) the type and concentration of organic acids. Inductively coupled plasma-atomic emission spectrometry (700 Series ICP OES, Agilent Technologies) was used to determine the concentrations of Ca, Mg, Fe, and Al. The pH was measured with a pH meter (DL70 ES, Mettler). The presence and concentration of the main organic acids released by strain PMB3(1) in solution were determined on an ion chromatography with conductivity detection (ICS 3000; Dionex Corp.) associated with an analytical column (IonPac AS 11 HC, Dionex Corp.) according to Balland et al. (75). Sodium formate, D-gluconic acid, sodium butyrate, pyruvic acid sodium salt, sodium citrate tribasic, sodium oxalate, sodium propionate, sodium acetate, succinic acid disodium salt, DL-malic acid, disodium salt, sodium-L-lactate, and malonic acid disodium salt were used as references. A subsample of the biotite particles (100 mg) remaining in each microcosm was recovered and washed 2 times with 2 mL sterile water to determine whether bacteria developed a strong interaction with mineral surfaces using a BX41 Olympus microscope at a magnification of 1,000×.

### RNA extraction

After 20 h of incubation, a volume of 10 mL of culture was recovered and centrifuged (8,000 × *g* 15 min at 4°C) to pellet the bacterial cells of each replicate. Cell pellets were frozen in liquid nitrogen and stored at −80°C. Total RNA was purified using the RiboPure kit (Ambion, Austin, USA) following the manufacturer’s recommendations, then treated with DNAse I (Ambion), precipitated with ethanol, and resuspended in RNAse-free water. The concentration and quality of the extracted RNA were determined using a Nanodrop 2000 spectrophotometer (Thermo Scientific, Waltham, MA, USA), a Qubit BR RNA Assay Kit in a Qubit 2.0 Fluorometer (Life Technologies, CA, USA), and TapeStation analyses. The RNA integrity number value was 9.0 on average. The total RNAs were sent to GENEWIZ, which operated the rRNA depletion using the Ribo-Zero rRNA removal kit for bacteria and performed the stranded RNA library preparation. The cDNA was sequenced on their Illumina NovaSeq platform using a 2 × 150 bp paired-end (PE) configuration. This sequencing generated a total of more than 234 million reads, ranging from 16 to 21 million per sample.

### Protein extraction and tandem mass spectrometry

A volume of 5 mL of culture was recovered and centrifuged to pellet the bacteria for each replicate. Supernatants were removed and cells were frozen in liquid nitrogen and stored at −80°C. For each sample, the cell pellet was homogenized in a given volume (25 µL per mg of cellular pellet, wet weight) of lithium dodecyl sulfate 1× lysis buffer (Invitrogen), supplemented with 200 mg of 0.1 mm silica beads (MP Biomedicals), and disrupted by bead-beating with a Precellys Evolution instrument (Bertin Technologies) operated at 7,800 rpm for three cycles of 20 s, with 30 s of pause between each cycle. The samples were then centrifuged at 16,000 *× g* for 1 min. The resulting supernatants were heated at 99°C for 5 min. A volume of 25 µL of each sample was loaded on a NuPAGE 4%–12% Bis-Tris gel and subjected to electrophoresis for 5 min at 200 V. After migration, the proteins were stained with Coomassie SimplyBlue SafeStain (Thermo) and destained with water. In-gel proteolysis of the whole proteome sliced in a single polyacrylamide band was performed with 0.2 µg of trypsin gold (Promega) supplemented with 50 µL of 0.01% ProteaseMAX surfactant (Promega) as previously described (76). After incubation at 37°C for 4 h, the solution was acidified with 5% trifluoroacetic acid and the final volume adjusted to 50 µL of 0.1% trifluoroacetic acid if required. A volume of 5 µL of this suspension was then injected to identify the resulting peptides by tandem mass spectrometry using a Q-Exactive HF instrument (Thermo Scientific) coupled to an Ultimate 300 nanoLC system (Thermo Scientific) and operated in a data-dependent acquisition mode in the same conditions as those described by Hayoun et al. (77). Peptides were resolved on an Acclaim PepMap100 C18 reversed-phase column (3 µm, 100 Å, 75 µm id ×50 cm) over a 90 min gradient.

### Statistical and bioinformatic analyses

The effect of the different treatments on the organic acid production, the pH, and inorganic nutrients released in solution, and the cell counts were determined by analysis of variance (one-way ANOVA, *P* < 0.05, followed by a Tukey test). Cell counts were first log-transformed before statistical analyses.

RNAseq reads were mapped to the coding sequences (CDS) of the genome of strain PMB3(1) through the MICROSCOPE (78) annotation platform using the BWA (version 0.7.4-r385) (79). The DESeq2 package (80, 81) was used to calculate differential gene expression abundance between the different treatments. A normalization of read numbers for replicates in each treatment was performed (by estimates of size factors in DESeq2 and normalization using the default parameters), then differential expression values (FoldChange) and statistical values (pVal and adjusted pVal through the Benjamini and Hochberg’s approaches), assessing the false discovery rate (FDR) were calculated. All genes having an adjusted *P*-value less than 0.05, a base mean score ≥150 a cut-off for absolute fold change ≥0.9 were considered as significantly differentially expressed. The genes differentially expressed were sorted according to the genome sequence of strain PMB3(1) (72) WXXL00000000), and raw data statistics are accessible on the SRA platform (BioProject: PRJNA787782, SRR17463930–SRR17463941, SRR32410786–SRR32410793).

For proteins, MS/MS spectra were queried against a database with the theoretical coding sequences of *Collimonas pratensis* PMB3(1) using the Mascot Daemon software version 2.6.1 (Matrix Science). The parameters used for this search were peptide charge of 2 + or 3+, 5 ppm peptide tolerance, 0.02 Da MS/MS fragment tolerance, carbamidomethylation of cysteine as a fixed modification, and oxidation of methionine as a variable modification. A protein was validated on the basis of two identified peptide sequences and with a final discovery rate of less than 1% as estimated with a decoy database search. The mass spectrometry proteomics data have been deposited to the ProteomeXchange Consortium via the PRIDE partner repository with the data set identifier PXD061836 and 10.6019/PXD061836. The DESeq2 package was also used to analyze the proteomics data using the same criteria described above, except that a cut-off for fold change >0.5 was considered.

A combined analysis of the RNA and protein data sets was done using the mixOmics R package (82, 83). For each approach (i.e., RNA or protein), data sets were first filtered to only retain consistent genes (i.e., with at least 100 in one sample for the RNAseq data set and at least 30 in the whole proteome data set). For each analysis, all the replicates have been considered, and default parameters have been used. A Partial Least Squares (PLS) regression was first done to integrate the RNAseq and proteome matrix of the BP vs BBP vs BCP treatments to generate a global view. PLS is a robust multivariate projection-based method used to explore or explain the relationship between two continuous data sets. To allow interpretability, a Sparse Partial Least Squares regression (sPLS) was then performed as it permits performing simultaneous variable selection. The distribution of the different genes considered was then visualized in a correlation circle plot, permitting the evidence of treatment-specific genes. The list of genes was then compared to the outputs of the DESeq2 analysis.

## Data Availability

The data that support the findings of this study are available on NCBI (BioProject PRJNA787782, SRR17463930-SRR17463941, and SRR32410786-SRR32410793) and PRIDE (PXD061836 and 10.6019/PXD061836). The chemical analyses are available from the corresponding authors on reasonable request.

## SUPPLEMENTAL MATERIAL

Supplemental figures aem.00704-25-s0001.docx Fig. S1 and S2.

Table S1 aem.00704-25-s0002.xlsx Common genes between BCPvsBBBP and BPvsBBP comparisons.

ASM does not own the copyrights to Supplemental Material that may be linked to, or accessed through, an article. The authors have granted ASM a non-exclusive, world-wide license to publish the Supplemental Material files. Please contact the corresponding author directly for reuse.
