# Supplementary material for: Decoding Collimonas pratensis PMB3(1) responses during biotite interaction and dissolution: a multi-omics and geochemical perspective
Source: Appl Environ Microbiol. 2025 Sep 19;91(10):e00704-25. doi: 10.1128/aem.00704-25 (PMC12542777; doi:10.1128/aem.00704-25)

**Supplementary materials:**

**TITLE**: Decoding *Collimonas pratensis* PMB3(1) responses during biotite interaction and dissolution: A multi-omics and geochemical perspective

**Running title:** Collimonas responses during biotite weathering

**AUTHORS :** Laura Picard^1,2^, Marie-Pierre Turpault^2^, Jean Armengaud^3^, Stéphane Uroz^1,2*^

^1^Université de Lorraine, INRAE, IAM, F- 54000 Nancy, France

^2^INRAE, BEF, Champenoux, F- 54000 Nancy France

^3^ CEA, INRAE, Département Médicaments et Technologies pour la Santé (DMTS), Université Paris Saclay, SPI, 30200 Bagnols-sur-Cèze, France

*** Corresponding author:** Mailing address: [stephane.uroz@inrae.fr](mailto:stephane.uroz@inrae.fr)

Université de Lorraine, INRAE, IAM, F-54000, Nancy, France.

Phone: +33 (0)3 83 39 40 57, Fax: +33 (0)3 83 39 40 69.

**Figure S1 : Experimental design and associated analyses.** Incubations were done in the BHm medium deprived or not in Fe and Mg. A total of six treatments was considered with 2 of them including the presence of a mineral (biotite) and a last four without mineral. Each flask was inoculated or not. The inoculum (*) corresponded to calibrated suspensions of the strain PMB3(1). Cell density was determined by measuring absorbance at 595nm and dilution/plating. The pH of the solution, the solution chemistry (cations, P) as well as the organic acids and the siderophore activity were determined after removal of bacterial cells and biotite particles and filtration at 0.22µm.


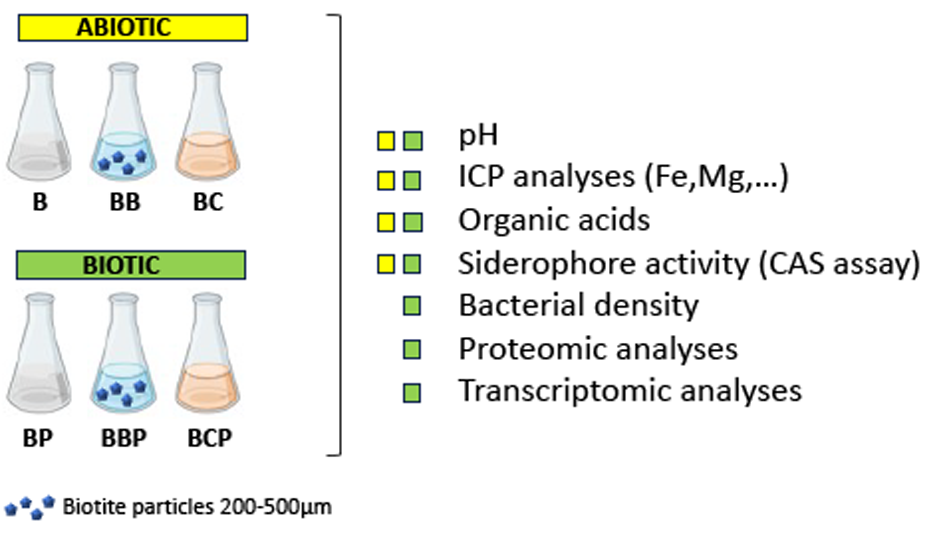


**Figure S2 : Proportions and Gene Ontology classifications of differentially expressed genes (DEGs) and differentially produced proteins (DAPs).** Comparison BP versus BCP (RNA, A; Protein, D), comparison of BBP versus BCP (RNA, B; protein, E) and comparison of BP versus BBP (RNA, C; protein, F). The detail of the COG categories is presented. A: RNA processing and modification (INFORMATION STORAGE AND PROCESSING); B: chromatin structure and dynamics (INFORMATION STORAGE AND PROCESSING); C: energy production and conversion (METABOLISM); D: Cell cycle control, cell division, chromosome partitioning (CELLULAR PROCESSES AND SIGNALLING); E: amino acid transport and metabolism (METABOLISM); F: nucleotide transport and metabolism (METABOLISM); G: carbohydrate transport and metabolism (METABOLISM); H: coenzyme transport and metabolism (METABOLISM); I: lipid transport and metabolism (METABOLISM); J: translation, ribosomal structure and biogenesis (INFORMATION STORAGE AND PROCESSING); K: transcription (INFORMATION STORAGE AND PROCESSING); L: replication, recombination and repair (INFORMATION STORAGE AND PROCESSING); M: cell wall/membrane/envelope biogenesis (CELLULAR PROCESSES AND SIGNALLING); N: Cell motility (CELLULAR PROCESSES AND SIGNALLING); O: Post-translational modification, protein turnover, chaperones (CELLULAR PROCESSES AND SIGNALLING); P: inorganic ion transport and metabolism (METABOLISM); Q: secondary metabolites biosynthesis, transport and catabolism (METABOLISM); R: general function prediction only (POORLY CHARACTERIZED); S: function unknown (POORLY CHARACTERIZED); T: signal transduction mechanisms (CELLULAR PROCESSES AND SIGNALLING); U: intracellular trafficking, secretion, and vesicular transport (CELLULAR PROCESSES AND SIGNALLING); V: defence mechanisms (CELLULAR PROCESSES AND SIGNALLING); W: Extracellular structures (

CELLULAR PROCESSES AND SIGNALLING)


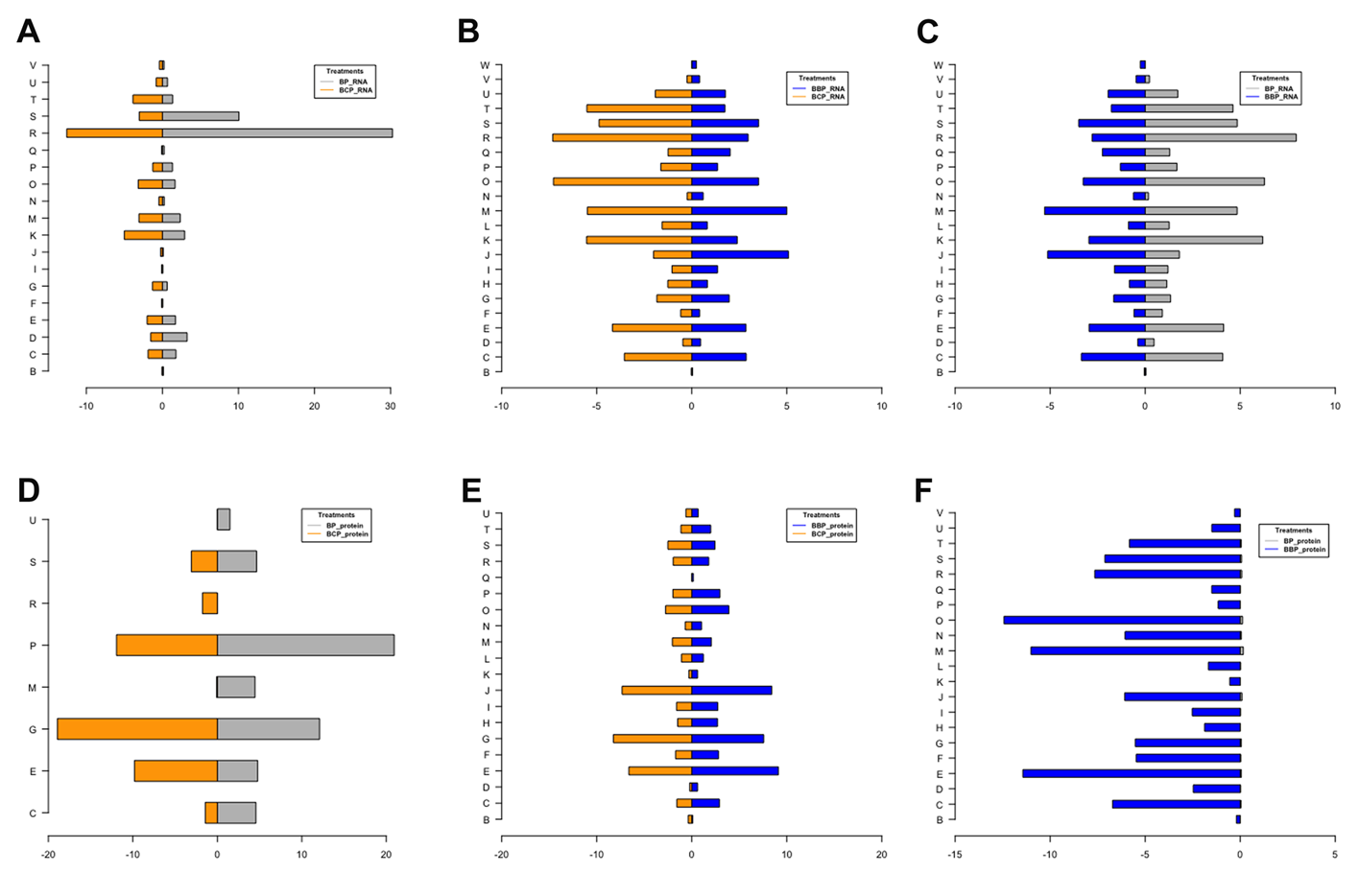

Supplement: Supplemental figures — Fig. S1 and S2. [file aem.00704-25-s0001.docx]
